# Sinomenine Hydrochloride Inhibits the Metastasis of Human Glioblastoma Cells by Suppressing the Expression of Matrix Metalloproteinase-2/-9 and Reversing the Endogenous and Exogenous Epithelial-Mesenchymal Transition

**DOI:** 10.3390/ijms19030844

**Published:** 2018-03-14

**Authors:** Yumao Jiang, Yue Jiao, Yang Liu, Meiyu Zhang, Zhiguo Wang, Yujuan Li, Tao Li, Xiaoliang Zhao, Danqiao Wang

**Affiliations:** Beijing Key Laboratory of Traditional Chinese Medicine Basic Research on Prevention and Treatment of Major Diseases, Experimental Research Center, China Academy of Chinese Medical Sciences, Beijing 100000, China; 13683301305@163.com (Y.J.); Jiaoyue_medicine@163.com (Y.J.); echoinapril@163.com (Y.L.); meiyu96@126.com (M.Z.); zhgw68_8@tom.com (Z.W.); lyjdcl@163.com (Y.L.); hndxlitao@163.com (T.L.); Zhaoxiaoliang1218@aliyun.com (X.Z.)

**Keywords:** glioblastoma, sinomenine hydrochloride, metastasis, matrix metalloproteinase, epithelial-mesenchymal transition

## Abstract

As shown in our previous study, sinomenine hydrochloride (SH), the major bioactive alkaloid isolated from *Sinomenium acutum* Rehd. et Wils. (Fam. *Menispermaceae*), initiates the autophagy-mediated death of human glioblastoma cells by generating reactive oxygen species and activating the autophagy-lysosome pathway. However, its effects on the migration and invasion of human glioblastoma cells have not yet been elucidated. Therefore, human glioblastoma U87 and SF767 cells were treated with SH (0.125 and 0.25 mM) for 24 h, and cell migration and invasion were assessed using scratch wound healing, migration and invasion assays. SH promoted G0/G1 phase arrest, inhibited the migration and invasion of the two cell lines, suppressed the activation of nuclear factor kappa B (NFκB) and the expression of matrix metalloproteinase (MMP)-2/-9, triggered endoplasmic reticulum (ER) stress, reversed the exogenous epithelial-mesenchymal transition (EMT) induced by the inflammatory microenvironment and the endogenous EMT. Additionally, NFκB p65 overexpression blocked the SH-mediated inhibitory effects on MMP-2/-9 expression and cell invasion. SH-induced autophagy was reduced in CCAAT/enhancer binding protein (C/EBP) homologous protein (CHOP) or autophagy-related 5 (ATG5)-silenced human glioblastoma cells and cells treated with 4-phenylbutyric acid (4-PBA) or 3-methyladenine (3-MA), as shown by the decreased levels of the microtubule-associated protein light chain 3B (LC3B)-II and autophagic vacuoles (AVs) stained with monodansylcadaverine (MDC), respectively. Moreover, knockdown of CHOP or ATG5 and treatment with 4-PBA or 3-MA abolished the SH-mediated inhibition of mesenchymal markers (vimentin, Snail and Slug) expression and cell invasion, respectively. Importantly, SH also regulated the above related pathways in nude mice. Based on these findings, SH inhibited cell proliferation by inducing cell cycle arrest, and attenuated the metastasis of U87 and SF767 cells by suppressing MMP-2/-9 expression and reversing the endogenous and exogenous EMT in vitro and/or in vivo. Thus, SH might be a new potential anti-metastasis agent for the treatment of human glioblastoma.

## 1. Introduction

Glioblastoma, a most common primary tumor of the central nervous system, shows substantial migration and invasion, resulting in frequent metastases into the surrounding tissues [1]. Because of its highly invasive nature, patients with glioblastoma often have a poor prognosis [2]. Therefore, the identification of potentially novel therapeutic agents that effectively inhibit human glioblastoma cell metastasis is urgently needed.

Matrix metalloproteinases (MMPs) play a central role in the invasion process by degrading many elements of the extracellular matrix (ECM), including collagens, fibronectin and laminins, and suppression of MMP-2/-9 expression represents a potential strategy for preventing tumor cell invasion [3,4]. Moreover, phorbol 12-myristate 13-acetate-induced MMP-9 expression is regulated by nuclear factor kappa B (NFκB) in human breast cancer cells [5]. NFκB is one of the major components of the intracellular signaling pathways responsible for MMP-2/-9 activation [6]. Additionally, NFκB activation is associated with cancer cell proliferation, angiogenesis, survival and invasion [7], and NFκB inactivation may be effective in treating and preventing cancer [8]. Although sinomenine has recently been reported to inhibit NFκB activation [9] or suppress MMP-2/-9 expression [10] to abrogate the invasiveness of various types of cancer cells, researchers have not determined whether sinomenine hydrochloride (SH) suppresses human glioblastoma cell metastasis through related mechanisms.

Autophagy is a cellular process that induces the recycling of cytoplasmic macromolecules and structures via the formation of double or multi-membrane-bound vacuoles called autophagosomes that degrade and engulf large portions of cells [11,12]. This process has been reported to exert both anti- and pro-metastatic effects, which may be context-dependent in cancer cells [13]. Knockdown of the autophagy regulators autophagy-related 3 (ATG3), ATG5 or ATG7 stimulates the migration of mouse embryonic fibroblast (MEF) and HeLa cells [14], and autophagy activation is associated with the degradation of Snail, a protein that regulates epithelial-mesenchymal transition (EMT) in breast cancer models [15]. The EMT and its reverse process (MET) are both essential for migration and invasion [16,17,18,19]. Specifically, EMT plays an important role in tumor dissemination and spreading. Once the EMT program is activated, tumor cells obtain an invasive phenotype that allows them to invade surrounding tissues and blood vessels, and then to detach from the primary site. Additionally, autophagy has been reported to be an adaptive response under endoplasmic reticulum (ER) stress conditions [20,21]. During ER stress, misfolded proteins accumulate in the ER and cannot be degraded by the proteasomal pathway, resulting in the upregulation of the unfolded protein response (UPR) and the increased expression of autophagy-related genes [22,23]. Moreover, ER stress responses have recently been shown to inhibit tumor cell migration and invasion [24,25]. Although our previous study reported that the anti-tumor effects of SH on human glioblastoma are mediated by autophagy activation [26], we have not determined whether ER stress is involved in SH-induced autophagy activation.

Metastasis mediated by inflammation is regarded as a great challenge during cancer therapy [27]. The inflammatory microenvironment promotes EMT progression to facilitate metastasis [28,29]. Based on accumulating data, macrophages promote metastasis in the tumor microenvironment [30] and release pro-inflammatory cytokines, including interleukin-1β (IL-1β) and tumor necrosis factor-α (TNF-α), to initiate inflammatory responses and subsequently induce tumor migration and invasion by promoting EMT through the regulation of transcription factors, such as Snail, Slug and Twist [31,32,33]. In recent years, several novel therapeutic interventions focusing on tumor invasion that target the EMT mediated by pro-inflammatory cytokines have been proposed. For instance, gemifloxacin inhibits TNF-α-mediated migration and EMT [34]. However, researchers have not clearly determined whether SH, a drug with anti-inflammatory effects, inhibits tumor cell metastasis induced by the inflammatory microenvironment. Based on the aforementioned observations, we hypothesized that SH suppresses human glioblastoma cell migration and invasion by regulating these factors.

SH is a hydrochloride form of sinomenine, the major bioactive alkaloid isolated from a traditional Chinese medicinal plant, *Sinomenium acutum* Rehd. et Wils. (Fam. Menispermaceae) and is widely used in the clinic to treat rheumatoid diseases due to its anti-inflammatory and anti-immune effects [35]. Sinomenine exerts its potent anticancer effects by enhancing apoptosis and autophagy, blocking metastasis, normalizing angiogenesis, overcoming drug resistance and coordinating with chemotherapeutic drugs; these effects have recently attracted increasing attention [26,36]. As shown in our previous study, SH induces autophagy-mediated death of human glioblastoma cells [26]. However, the effect of SH on the metastasis of human glioblastoma cells is still unknown. Therefore, the aim of this study was to investigate whether SH exerts inhibitory effects on human glioblastoma cell metastasis and to explore its potential mechanisms of action. Our results revealed that SH inhibited proliferation by inducing cell cycle arrest and attenuated the metastasis of human glioblastoma U87 and SF767 cells by suppressing the expression of MMP-2/-9 and reversing endogenous and exogenous EMT in vitro and/or in vivo.

## 2. Results

### 2.1. Sinomenine Hydrochloride (SH) Selectively Kills Human Glioblastoma Cells, But Not Normal Glial Cells, and Induces Human Glioblastoma Cell Cycle Arrest

**Figure 1:**
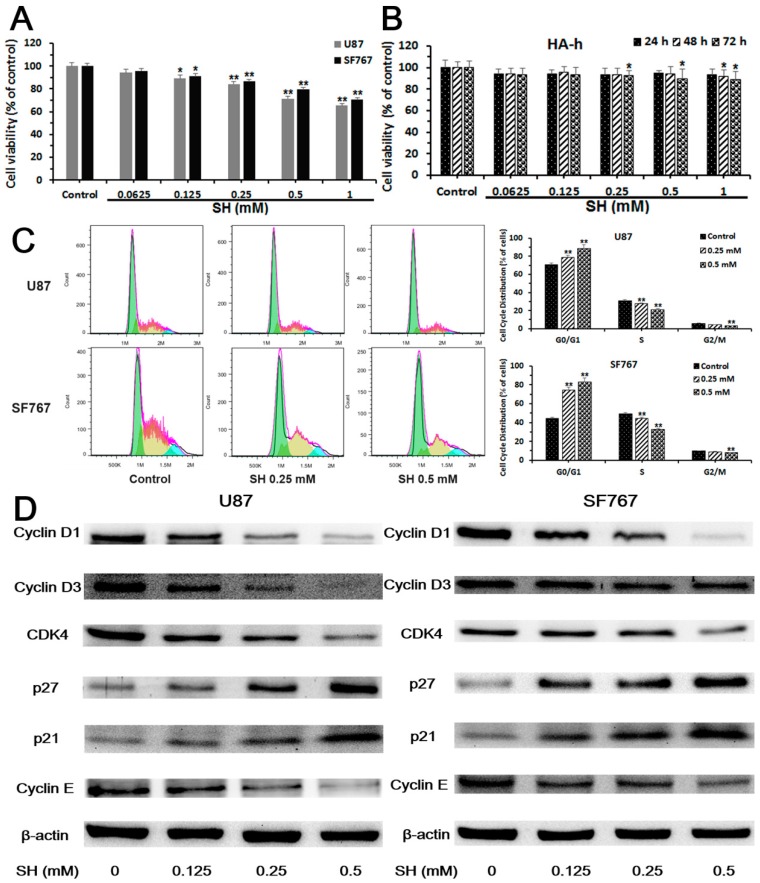
Sinomenine hydrochloride (SH) selectively kills human glioblastoma cells, but not normal glial cells, and induces human glioblastoma cell cycle arrest. (**A**) The human glioblastoma cell lines were treated with SH (0.0625 to 1.0 mM) for 24 h, and cell counting kit-8 (CCK-8) assays were applied to analyze cell viability; (**B**) HA-h cells were treated with SH (0.0625 to 1.0 mM) for the indicated time points, and CCK-8 assays were used to examine cell viability; (**C**) Analysis of the DNA content and histograms of the cell cycle phase distribution of U87 and SF767 cells treated with SH (0.25, 0.5 mM) for 24 h; (**D**) The indicated concentrations of SH dose-dependently altered the levels of cell cycle-related proteins in U87 and SF767 cells at 24 h. Each blot and image shown is representative of *n* = 3 experiments. All data are presented as means ± SEM, *n* = 3. * *p* < 0.05, ** *p* < 0.01 compared with the control.

We assessed the viability of human glioblastoma U87 and SF767 cells incubated with various concentrations of SH (0.0625, 0.125, 0.25, 0.5 and 1.0 mM) for 24 h using cell counting kit-8 (CCK-8) assays to evaluate the effect of SH on cell proliferation. As shown in Figure 1A, SH did not exert a significant cytotoxic effect on cell proliferation at 0.0625, 0.125 and 0.25 mM, although higher concentrations of SH (0.5 and 1.0 mM) produced apparent cytotoxic effects on U87 and SF767 cells at 24 h, which were mentioned in our previous study [26]. Therefore, we used SH concentrations ranging between 0.0625 and 0.25 mM to avoid the inhibition of cell viability in experiments assessing the anti-metastasis effects of SH. In addition, human astrocyte-hippocampal (HA-h) cells were chosen to examine the selective toxicity of SH. As shown in Figure 1B, SH exerted stronger toxic effects on neoplastic cells than HA-h cells.

Additionally, we observed the effect of SH on the cell cycle distribution using propidium iodide (PI) staining to investigate whether SH mediated cell cycle arrest. As shown in Figure 1C, cells were arrested at G0/G1 phase. The increased number of cells in G0/G1 phase after SH treatment was associated with a reduced number of cells in G2/M and S phases compared to the control. We examined the levels of cell cycle-related proteins, including cyclin D1, cyclin D3, cyclin E and cyclin-dependent kinase 4 (CDK4), in U87 and SF767 cells to clarify the molecular mechanisms by which SH mediated G0/G1 phase arrest. Compared with control cells, SH-treated cells exhibited dose-dependent decreases in the levels of cyclin D1, cyclin D3, cyclin E and CDK4 (Figure 1D), consistent with the functions of these proteins in regulating the G0/G1 phase transition; meanwhile, we analyzed the effect of SH on the levels of critical regulators of G0/G1 phase progression, including the CDK inhibitors p27Kip1 and p21Waf1/Cip1 [37,38]. As shown in the Western blots presented in Figure 1D, the SH treatment dose-dependently upregulated p27 and p21 expression, indicating that SH elevates the levels of CDK inhibitors, which in turn mediate G0/G1 phase arrest.

### 2.2. SH Inhibits the Migration and Invasion of U87 and SF767 Cells

**Figure 2:**
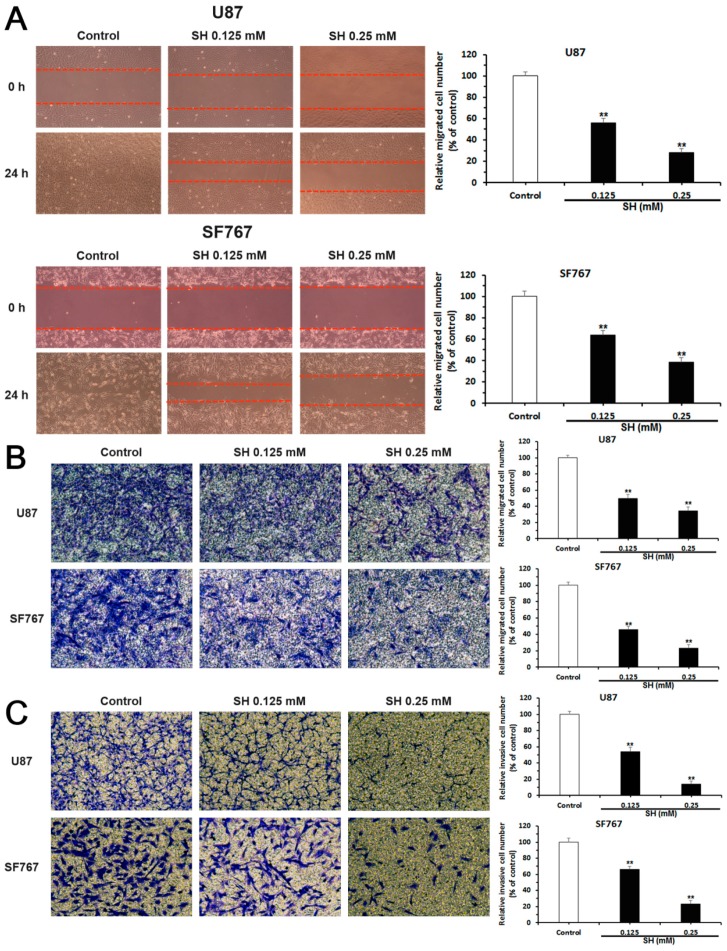
Effects of SH on the migration and invasion of human glioblastoma cells in vitro. U87 and SF767 cells were treated with SH (0.125, 0.25 mM) for 24 h. (**A**) Results and statistical analysis of wound healing assays for U87 and SF767 cells. The red dotted lines represent the locations of cell migration to the wounded area; (**B**) Results and statistical analysis of Transwell migration assays for U87 and SF767 cells; and (**C**) results and statistical analysis of matrigel-coated Transwell invasion assays for U87 and SF767 cells. All images were captured at 100× magnification. Each image is representative of *n* = 3 experiments. All data are presented as means ± SEM, *n* = 3. ** *p* < 0.01 compared with the control.

We detected the effects of SH on human glioblastoma cell metastasis using scratch wound healing assays, Transwell migration assays and matrigel-coated Transwell invasion assays. As shown in Figure 2A, in the SH (0.125 and 0.25 mM)-treated groups, fewer cells migrated to the wounded zone compared with the control U87 and SF767 cells at 24 h, and Transwell migration assays also showed a significant decrease in the migration of U87 and SF767 cells following treatment with SH (0.125 and 0.25 mM) compared with the control group at 24 h (Figure 2B). We then investigated the effects of SH on the invasion of human glioblastoma cells using matrigel-coated Transwell invasion assays. As shown in Figure 2C, the invasion of U87 and SF767 cells was markedly attenuated following treatment with SH (0.125 and 0.25 mM) compared with the control group at 24 h. Thus, SH decreases the invasion of the human glioblastoma cells in a dose-dependent manner, consistent with the inhibitory effects of SH on the migration of the two cell lines.

### 2.3. SH Decreases MMP-2/-9 Expression by Suppressing NFκB Activation

**Figure 3:**
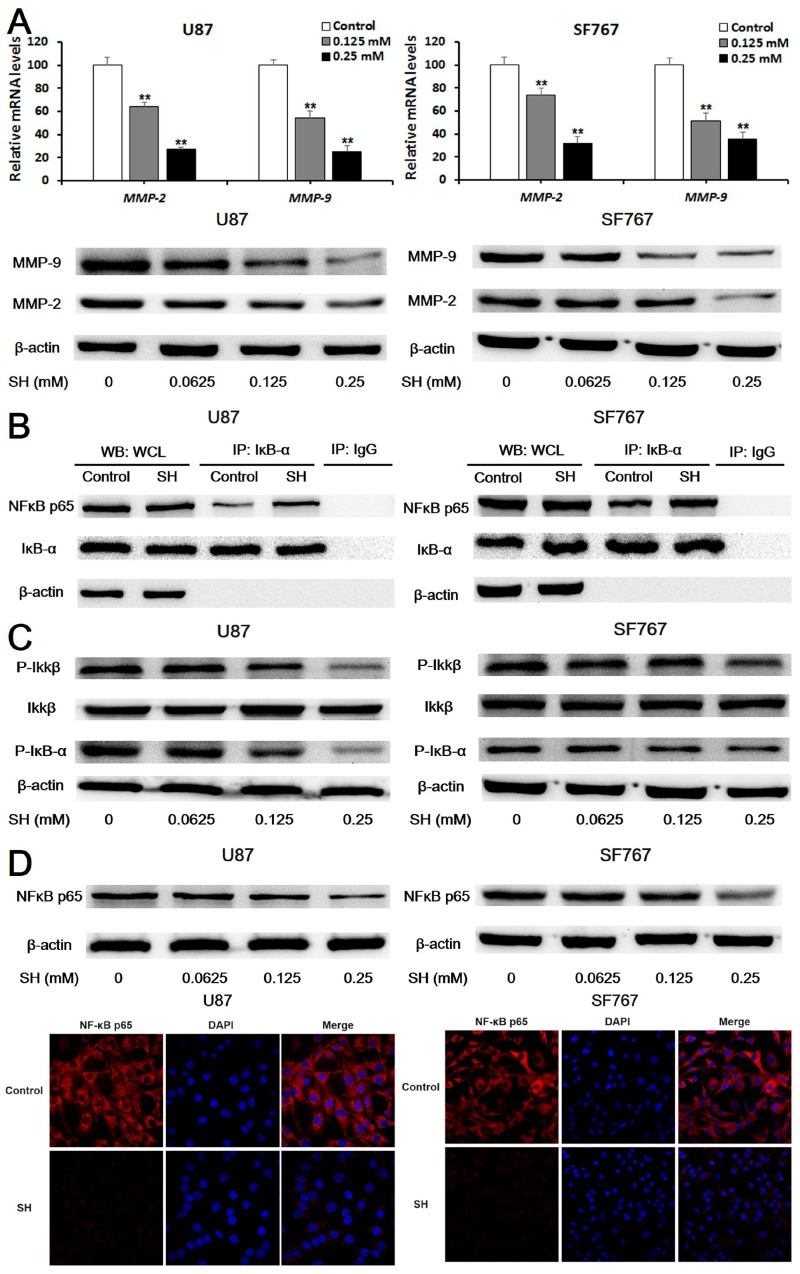

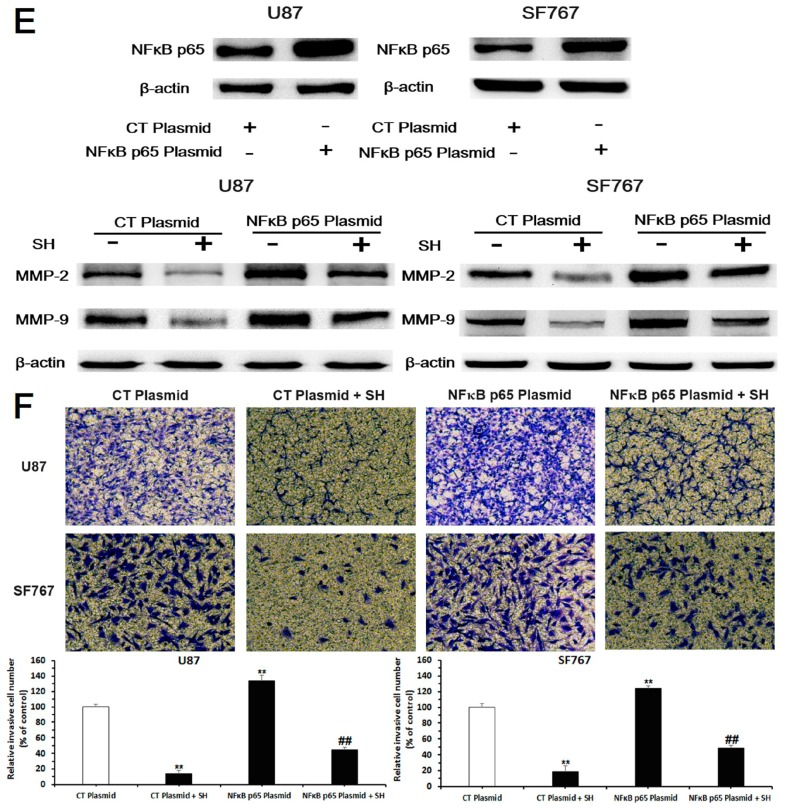
SH reduced matrix metalloproteinase (MMP)-2/-9 expression by suppressing nuclear factor kappa B (NFκB) activation. (**A**) After treatment with the indicated concentrations of SH for 24 h, levels of the *MMP-2/-9* mRNAs in U87 and SF767 cells were assessed by quantitative real-time PCR, and levels of the MMP-2/-9 proteins in the two cell lines were examined using Western blot analysis; (**B**) Co-immunoprecipitation (Co-IP) analysis of the binding of NFκB p65 and inhibitor of NFκB (IκB)-α. Cells were incubated with SH (0.25 mM) for 6 h. An IκB-α antibody was used in the IP experiment, an NFκB p65 antibody was used in the subsequent Western blot analysis, and an IgG antibody was applied as the negative control. WCL: whole cell lysates; (**C**) The SH treatment decreased levels of phosphorylated inhibitor kappa B kinase (IKK) β and IκB-α. U87 and SF767 cells were incubated with the indicated concentrations of SH for 24 h; (**D**) SH inhibited NFκB p65 expression in U87 and SF767 cells. Prior to the Western blot analysis, cells were treated with the indicated concentrations of SH for 24 h. For the immunofluorescence assays, cells were treated with SH (0.25 mM) for 24 h, fixed, stained with primary antibodies against NFκB p65, and stained with fluorescein isothiocyanate (FITC)-labeled secondary antibodies (red fluorescence). Nuclei were stained with diamidino-phenyl-indole (DAPI) (blue fluorescence). The protein was observed using a laser scanning confocal microscope, the images of U87 cells were captured at 1000× magnification and the images of SF767 cells were captured at 400× magnification; (**E**) Efficiency of NFκB p65 overexpression and the effects of an NFκB p65 plasmid on SH-induced down-regulation of MMP-2/-9 expression. Cells were transfected with the no-target control plasmid (CT plasmid) or the NFκB p65 plasmid for 24 h and then treated with or without SH (0.25 mM) for another 24 h; (**F**) Effects of the NFκB p65 plasmid on the SH-mediated reduced invasion of the two cell lines in matrigel-coated Transwell invasion assays (100× magnification). Cells were transfected with the CT plasmid or the NFκB p65 plasmid for 24 h and then treated with or without SH (0.25 mM) for an additional 24 h. Each image is representative of *n* = 3 experiments. All blots shown above are representative of *n* = 3 experiments with similar results. β-Actin served as the loading control. All data are presented as means ± SEM, *n* = 3. ** *p* < 0.01 compared with the control; ^##^ *p* < 0.01 compared with the NFκB p65 plasmid-transfected group.

First, we determined whether SH regulated MMP-2/-9 expression. As shown in Figure 3A, we evaluated levels of the *MMP-2/-9* mRNAs and MMP-2/-9 proteins. Based on the quantitative real-time PCR results, treatment with SH (0.125 and 0.25 mM) for 24 h markedly down-regulated the levels of *MMP-2/-9* mRNAs in U87 and SF767 cells. Additionally, the indicated concentrations of SH reduced the levels of MMP-2/-9 proteins in the two cell lines in a dose-dependent manner at 24 h, as evidenced by Western blot analysis. The above findings indicate that SH regulates the expression of MMP-2/-9 at the transcriptional and translational levels.

Second, we performed co-immunoprecipitation (co-IP) assays to detect the binding of NFκB p65 to the inhibitor of NFκB (IκB)-α in U87 and SF767 cells treated with SH (0.25 mM). According to the Western blot analysis, the binding of NFκB p65 to IκB-α was enhanced by the SH treatment (Figure 3B). Moreover, SH dose-dependently decreased the levels of phosphorylated inhibitor kappa B kinase (IKK) β and IκB-α (Figure 3C). Additionally, we examined cells treated with the indicated concentrations of SH for 24 h using Western blot analysis and photographed them with a laser scanning confocal microscope, as shown in Figure 3D, to visualize the effects of SH on NFκB p65 expression. SH decreased levels of the NFκB p65 protein in U87 and SF767 cells. Thus, the SH treatment inhibited NFκB activation in the two cell lines.

Third, we transfected the two cell lines with an NFκB p65 plasmid to assess whether the inhibitory effects of SH on MMP-2/-9 expression were associated with NFκB inactivation. Treatment with the combination of SH and the NFκB p65 plasmid reversed the inhibitory effects of SH on MMP-2/-9 expression (Figure 3E) and the invasion (Figure 3F) of U87 and SF767 cells.

Based on these observations, the NFκB/MMP pathway inhibition is involved in SH-mediated suppression of human glioblastoma cell invasion.

### 2.4. SH Impairs the Invasiveness of Human Glioblastoma Cells by Reversing the Endogenous EMT through ER Stress-Mediated Autophagy

We preformed the experiments listed below to determine if the SH-mediated inhibition of U87 and SF767 cell invasion is associated with the reversal of the EMT through ER stress-mediated autophagy.

**Figure 4:**
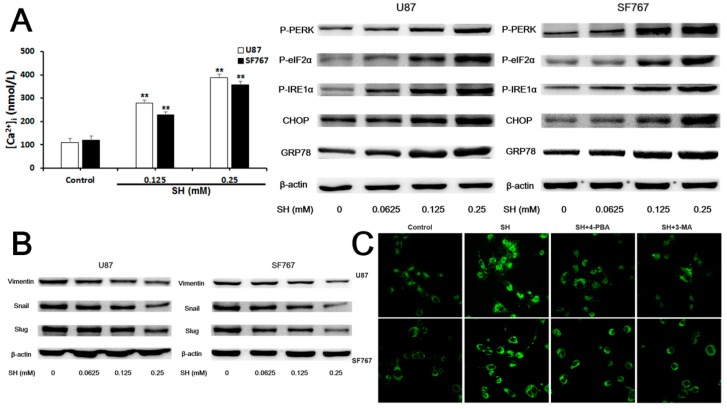

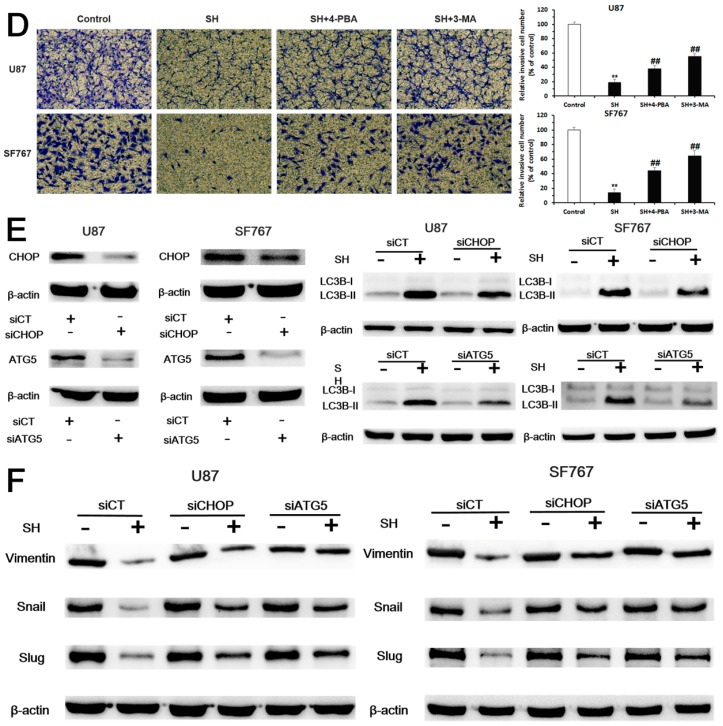
SH impairs the invasiveness of human glioblastoma cells by reversing epithelial-mesenchymal transition (EMT) through endoplasmic reticulum (ER) stress-mediated autophagy. (**A**) Effects of SH on [Ca^2+^]i and levels of ER stress-associated proteins in U87 and SF767 cells. Cells were treated with the indicated concentrations of SH for 24 h; (**B**) Effects of SH on the protein levels of mesenchymal markers in the two cell lines. Cells were treated with the indicated concentrations of SH for 24 h; (**C**) Twenty-four hours after SH (0.25 mM) treatment in the presence or absence of 4-phenylbutyric acid (4-PBA) or 3-methyladenine (3-MA), the two cell lines were stained with monodansylcadaverine (MDC) and photographed with a laser scanning confocal microscope (600× magnification); (**D**) Treatments with 4-PBA or 3-MA compensated for the SH (0.25 mM)-mediated inhibition of the invasiveness of the two cell lines at 24 h (100× magnification); (**E**) Efficiency of CCAAT/enhancer binding protein (C/EBP) homologous protein (CHOP) or autophagy-related 5 (ATG5) knockdown and the effects of CHOP or ATG5 silencing on the SH-induced increase in the microtubule-associated protein light chain 3B (LC3B)-II levels. Cells were transfected with a no-target control siRNA (siCT), CHOP siRNA (siCHOP) or ATG5 siRNA (siATG5) for 24 h and then treated with or without SH (0.25 mM) for an additional 24 h; (**F**) Effects of CHOP or ATG5 silencing on the SH-mediated decreased protein levels of mesenchymal markers. Cells were transfected with siCT, siCHOP or siATG5 for 24 h and then treated with or without SH (0.25 mM) for an additional 24 h. Each blot and image shown is representative of *n* = 3 experiments. Data represents means ± SEM, *n* = 3. ^##^ *p* < 0.01 compared with the SH treatment alone; ** *p* < 0.01 compared with the control.

First, as shown in Figure 4A, SH elevated the cytoplasmic concentrations of free Ca^2+^ and the levels of P-eukaryotic translation initiation factor 2α kinase 3 (PERK), P-eukaryotic translation initiation factor 2α (eIF2α), P-inositol-requiring enzyme 1 (IRE1) α, CCAAT/enhancer binding protein (C/EBP) homologous protein (CHOP) and ER intraluminal 78 kDa glucose-regulated protein (GRP78). Meanwhile, SH decreased the protein levels of mesenchymal markers, including vimentin, Snail and Slug (Figure 4B).

Second, compared to SH treatment, combined treatments with SH and the chemical ER stress inhibitor 4-phenylbutyric acid (4-PBA) or the autophagy inhibitor 3-methyladenine (3-MA) decreased the numbers of autophagic vacuoles (AVs) stained with monodansylcadaverine (MDC) (Figure 4C) and promoted the invasion of the two cell lines (Figure 4D).

Third, blockade of ER stress with a CHOP small interfering RNA (siRNA) or suppression of autophagy with an ATG5 siRNA reduced the levels of microtubule-associated protein light chain 3B (LC3B)-II, an autophagy marker, in response to SH (Figure 4E) and reversed the effects of SH on the protein levels of mesenchymal markers, including vimentin, Snail and Slug, in the two cell lines (Figure 4F).

Based on these observations, SH impaired the invasion of U87 and SF767 cells by reversing the EMT through ER stress-mediated autophagy.

### 2.5. SH Inhibits the EMT and Invasion of Human Glioblastoma Cells in the Inflammatory Microenvironment

#### 2.5.1. Establishment of an Inflammatory Microenvironment System

**Figure 5:**
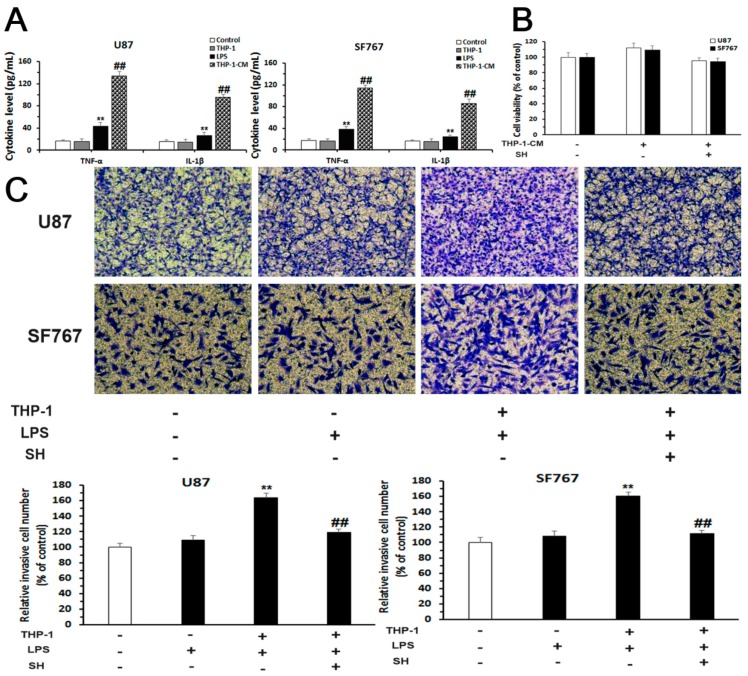

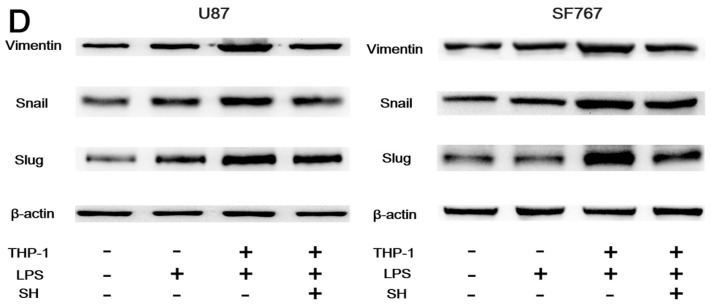
SH inhibits the EMT and invasion of the human glioblastoma cells in the inflammatory microenvironment. (**A**) Levels of the inflammatory cytokines tumor necrosis factor-α (TNF-α) and interleukin-1β (IL-1β) in the medium of U87 and SF767 cells that had been treated with lipopolysaccharides (LPS) (10 ng/mL), the medium of THP-1 cells or THP-1-CM for 24 h were assessed using enzyme-linked immunosorbent assays (ELISAs); (**B**) The viability of U87 and SF767 cells that had been treated with THP-1-CM or THP-1-CM containing SH (0.25 mM) for 24 h was investigated using CCK-8 assays; (**C**) Invasion assays were conducted using Transwell inserts covered with matrigel. Cells treated with LPS (10 ng/mL), THP-1-CM or THP-1-CM containing SH (0.25 mM) for 24 h were subjected to matrigel-coated Transwell invasion assays (100× magnification); (**D**) Western blot analysis was applied to investigate the levels of vimentin, Snail and Slug in cells incubated with LPS (10 ng/mL), THP-1-CM or THP-1-CM containing SH (0.25 mM) for 24 h. Each blot and image shown is representative of *n* = 3 experiments. β-Actin served as the loading control. All data are presented as means ± SEM, *n* = 3. ** *p* < 0.01 compared with the control; ^##^ *p* < 0.01 compared with the LPS- or THP-1-treated group for the results shown in A or ^##^ *p* < 0.01 compared with the THP-1-CM-treated group for the results shown in C. THP-1: the medium of THP-1 cells; THP-1-CM: the medium of THP-1 cells stimulated with LPS (10 ng/mL) for 24 h.

We treated U87 and SF767 cells with lipopolysaccharides (LPS) alone, the medium of THP-1 cells (a human acute monocytic leukemia cell line) alone or THP-1-conditioned-medium (THP-1-CM) for 24 h to establish pro-inflammatory conditions simulating the actual tumor microenvironment. As determined using enzyme-linked immunosorbent assays (ELISAs), the concentrations of TNF-α and IL-1β were significantly elevated in the two cell lines stimulated with THP-1-CM (the medium of THP-1 cells stimulated with 10 ng/mL LPS for 24 h) (Figure 5A). In the CCK-8 assays, THP-1-CM or THP-1-CM containing SH (0.25 mM) did not exert obvious cytotoxic effects on U87 and SF767 cell viability (Figure 5B). Thus, SH (0.25 mM) and THP-1-CM were chosen for further experiments.

#### 2.5.2. SH Abolishes THP-1-CM-Stimulated Exogenous EMT and Invasion in U87 and SF767 Cells

We then investigated the potential anti-invasive effect of SH. First, the effect of SH on cell invasion stimulated by THP-1-CM was assessed using matrigel-coated Transwell invasion assays. As shown in Figure 5C, SH suppressed the THP-1-CM-augmented invasion of U87 and SF767 cells.

Second, SH treatments prohibited the up-regulated expression of mesenchymal markers, including vimentin, Snail and Slug, mediated by THP-1-CM in the two cell lines (Figure 5D).

Thus, SH hinders the THP-1-CM-induced EMT and invasion of the two cell lines.

### 2.6. SH Regulates the Levels of Proliferation and Metastasis-Related Proteins in Glioblastoma Tumors In Vivo

**Figure 6:**
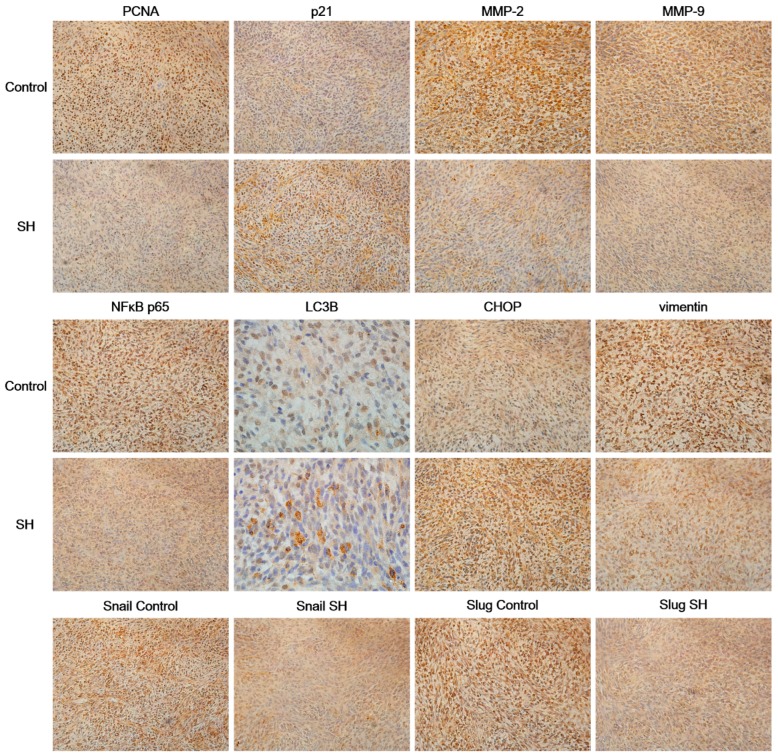
SH regulates the levels of proliferation and metastasis-related proteins in glioblastoma tumors in vivo. Immunohistochemical staining for proliferating cell nuclear antigen (PCNA), p21, MMP-2/-9, NFκB p65, LC3B, CHOP, vimentin, Snail and Slug in tumor tissue samples treated with SH (75 mg/kg) or physiological saline. The representative overview images of immunohistochemical staining for LC3B were captured at 1000× magnification; all the others were captured at 400× magnification. Each image is representative of *n* = 7 samples, with similar results.

We performed immunohistochemical staining of tumor tissues obtained from nude mice bearing U87 xenografts from our previous experiments [26] to further investigate the effect of SH on the proliferation and metastasis of tumor cells in vivo at the molecular level. Consistent with the results of the in vitro experiments, the immunohistochemical staining showed that SH treatment dramatically increased the levels of p21 and CHOP, increased the numbers of LC3B puncta and decreased the levels of proliferating cell nuclear antigen (PCNA), MMP-2/-9, NFκB p65, vimentin, Snail and Slug in tumor tissues compared to the control group (Figure 6). Based on these data, SH inhibits cell proliferation, induces cell cycle arrest, suppresses the expression of MMP-2/-9, inactivates NFκB, triggers ER stress and autophagy, and reverses EMT in vivo.

## 3. Discussion

Our study is the first to show that SH effectively suppresses the metastasis of human glioblastoma. This anticancer effect of SH is mediated by decreasing the MMP-2/-9 levels and reversing the endogenous and exogenous EMT in vitro and/or in vivo.

Cell invasion is a complex process related to the interaction of tumor cells with stromal cells and the ECM [39]. ECM degradation is involved in glioblastoma invasion into the normal brain parenchyma, and this process is accomplished by MMP secretion from cancer cells [40]. MMPs degrade the ECM and lead to the destruction of connective tissue barriers, such as collagens, fibronectin, laminins, vitronectin and heparan sulfate proteoglycans [41]. Thus, MMPs are considered crucial elements in tumor cell metastasis. Specifically, MMP-2/-9 are known as important enzymes in invasion, and their levels increase during glioblastoma progression [42,43]. In the present study, SH markedly reduced the levels of the MMP-2/-9 mRNAs and proteins and inhibited the metastasis of U87 and SF767 cells. Thus, the inhibitory effects of SH on glioblastoma cell metastasis are associated with reduced MMP-2/-9 expression. These findings are consistent with a previous report showing that the sinomenine treatment suppresses the migration and invasion of human osteosarcoma cells by inhibiting the expression of MMP-2/-9 [10]. Additionally, NFκB acts as a nuclear transcription factor for MMPs, and the promoter regions of MMP genes contain NFκB-responsive elements [44]. Therefore, NFκB plays a key role in cell invasion, and a variety of pathways regulate NFκB activation. Under general conditions, NFκB subunits combine with its inhibitory protein IκB in the cytoplasm. Under an active state, IκB kinase, P-IKKβ, phosphorylates IκB, resulting in proteasomal and ubiquitination degradation of IκB and the nuclear translocation of free NFκB dimers [45]. Here, the binding of NFκB p65 to IκB-α was enhanced by the SH treatment, and SH dose-dependently decreased the levels of phosphorylated IKKβ, phosphorylated IκB-α and the NFκB p65 protein in U87 and SF767 cells. Thus, SH inhibits NFκB activation through multiple mechanisms, meanwhile, NFκB p65 overexpression reversed the SH-mediated decrease in MMP-2/-9 levels and impaired the invasiveness of U87 and SF767 cells, consistent with previous results showing that sinomenine suppresses breast cancer cell metastasis by blocking NFκB activation [9]. Thus, SH hinders the invasion of human glioblastoma cells by inhibiting the NFκB/MMP pathway.

Autophagy is known as a mechanism to package organelles and protein complexes to degrade these cytoplasmic components and is thus critical for regulating cell internal homeostasis and growth [46]. The functions of autophagy in tumor metastasis are complicated, as studies have reported both anti-metastatic and pro-metastatic roles for autophagy. The stage specificity of tumor metastasis may influence the cellular response to autophagy [13]. Moreover, the use of different model systems (mouse or different human cell lines) may explain the obvious variation in results [15,47,48,49,50]. Nevertheless, autophagy activation has been shown to impair the metastasis of tumor cells by reversing EMT [47,51,52,53]. EMT is a process by which cells switch their epithelial properties to a mesenchymal and invasive phenotype, enabling them to invade the ECM [54,55]. At the molecular level, EMT induces the down-regulation of epithelial markers and the up-regulation of mesenchymal markers, including the intermediate filament protein vimentin and EMT-associated transcription factors (e.g., Snail, Slug and Twist) [54,55]. Here, autophagy exerted a direct effect on the mobility of glioblastoma cells. SH-mediated autophagy activation impaired the metastasis of U87 and SF767 cells. Moreover, in our model, down-regulation of vimentin, Snail and Slug was observed upon autophagy induction, consistent with the results of a previous study on glioblastoma cells [51]. Additionally, autophagy has often been regarded as a consequence of ER stress and is observed as an alternate pathway to remit ER stress or participate in cell death mediated by ER stress [21,56]. The ER is a specific organelle required for protein folding/maturation and Ca^2+^ storage. Under ER stress conditions, Ca^2+^ is released into the cytosol and triggers autophagy by activating the calcium/calmodulin-dependent protein kinase kinase (CaMKK)-adenosine 5′-monophosphate (AMP)-activated protein kinase (AMPK)-mammalian target of rapamycin (mTOR) signaling pathway [57]. Moreover, ER stress initiates a complicated intracellular signaling pathway called the UPR. The UPR reestablishes ER homeostasis through adaptive mechanisms involving the activation of autophagy, however, persistent ER stress can promote cell death through the UPR and autophagy [21,56]. Three major pathways are involved in UPR signaling: the PERK pathway, the IRE1 pathway, and the activating transcription factor 6 (ATF6) pathway [58]. Under normal conditions, the luminal domains of these sensor proteins interact with GRP78, an immunoglobulin-binding protein [59]. However, following the depletion of Ca^2+^ stores, the accumulation of misfolded proteins or glucose deprivation, GRP78 binding to misfolded and unfolded proteins is enhanced under ER stress conditions, resulting in its detachment from PERK, IRE1 and ATF6; this dissociation causes the release of these ER stress sensors and then activates the UPR [60]. PERK, whose intrinsic kinase activity is triggered by oligomerization, mediates the phosphorylation of eIF2α [61]. In response to persistent ER stress, the P-eIF2α, IRE1α and ATF6 signaling pathways increase the expression of CHOP, the major effector of the UPR or ER stress [62,63]. Activation of ER stress, along with the induction of autophagy, by a variety of chemotherapeutic agents was recently reported in tumor cells, although the mechanistic link between ER stress and autophagy has not been fully elucidated [21,64,65]. Consistent with these findings, in the present study, SH increased the cytoplasmic concentration of free Ca^2+^ and the expression levels of ER stress-related proteins. Moreover, the combined treatment with SH and 4-PBA or 3-MA strongly prevented the increased numbers of AVs and the inhibition of the invasion of the two cell lines mediated by SH, and the combined treatment with SH and the CHOP siRNA or ATG5 siRNA significantly reversed the effects of SH on the levels of the autophagy marker LC3B-II and mesenchymal markers, including vimentin, Snail and Slug, in both human glioblastoma cell lines. Based on these observations, SH impairs the invasiveness of the two cell lines by reversing the EMT through ER stress-mediated autophagy. Notably, EMT induction is associated with an intricate network of complex signal transduction pathways, and many studies have reported that NFκB activation induces invasive behaviors in different types of cells by regulating the EMT [66,67,68,69]. Thus, the SH-mediated inhibition of NFκB nuclear translocation may also be involved in the molecular mechanisms underlying the reversal of the EMT. Nevertheless, additional evidence is required to support this hypothesis.

The tumor microenvironment comprises actively infiltrating immune cells, and macrophages mediate the inflammatory responses via the release of pro-inflammatory cytokines. Because macrophages are a major source of pro-inflammatory cytokines, including TNF-α, IL-1β, IL-6 and IFN-γ, the medium of THP-1 monocytes stimulated with LPS was used to simulate the inflammatory microenvironment in the present study. Pro-inflammatory cytokines observed in the tumor microenvironment have been shown to promote metastasis, facilitating the effects on cancer cells by activating the EMT [31,32]. TNF-α and IL-1β levels were increased in U87 and SF767 cells stimulated with THP-1-CM; moreover, SH successfully abrogated the THP-1-CM-mediated increase in levels of mesenchymal markers, including vimentin, Snail and Slug, and the enhanced invasion of the two cell lines. Thus, SH inhibits the invasion of the human glioblastoma cells by reversing the exogenous EMT stimulated by the tumor inflammatory microenvironment. However, the precise underlying mechanisms still require further study.

**Figure 7:**
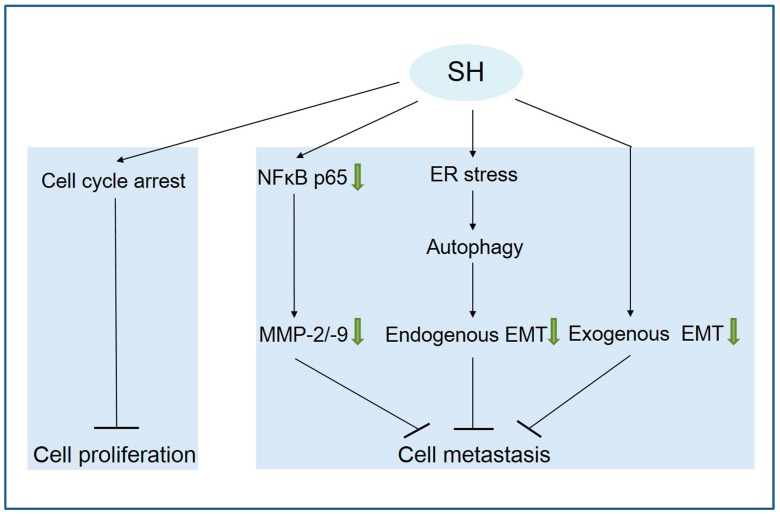
Proposed model delineating the mechanisms of SH in human glioblastoma cells. The green arrow means inhibition; “↓” means promotion; “┴” means inhibition.

In summary, this study is the first to report that SH inhibits human glioblastoma cell proliferation by inducing cell cycle arrest and attenuates the metastasis of U87 and SF767 cells through the following mechanisms (Figure 7): (1) suppressing the expression of MMP-2/-9 via NFκB inactivation; (2) reversing the endogenous EMT through ER stress-mediated autophagy; and (3) reversing the exogenous EMT stimulated by the tumor inflammatory microenvironment. Thus, SH represents a potentially novel candidate anti-metastasis agent for human glioblastoma therapy.

## 4. Materials and Methods

### 4.1. Reagents and Antibodies

SH was purchased from Zhengqing Pharmaceutical Group (Hunan, China). Diamidino-phenyl-indole (DAPI), 3-MA, 4-PBA, LPS and MDC were purchased from Sigma-Aldrich (St. Louis, MO, USA). CCK-8 was purchased from Dojindo Laboratories (Kumamoto, Japan). The Lipofectamine™ 2000 transfection reagent was purchased from Invitrogen (Life Technologies, Grand Island, NY, USA). Antibodies against PCNA, CDK4, p21, Cyclin D1, Cyclin D3, p27, Cyclin E, NFκB p65, phospho-IκB-α (Ser32), IκB-α, GRP78, phospho-PERK (Thr 981), ATG5, vimentin, Snail, Slug and β-actin were purchased from Santa Cruz Biotechnology (Santa Cruz, CA, USA). Antibodies against phospho-eIF2α (Ser51) and CHOP were purchased from Cell Signaling Technology (Danvers, MA, USA). Antibodies against phospho-IRE1α (Ser724), MMP-2, MMP-9, phospho-IKKβ (Tyr 188), IKKβ and LC3B were purchased from Abcam (Cambridge, UK). All secondary antibodies were purchased from Santa Cruz Biotechnology.

### 4.2. Cell Culture and Treatment

The human glioblastoma cell lines U87 and SF767, HA-h cells and human acute monocytic leukemia THP-1 cells were purchased from the Cell Resource Center of the Chinese Academy of Medical Science (Beijing, China) and were cultured in high glucose Dulbecco’s Modified Eagle’s Medium (DMEM-H) supplemented with penicillin (100 unit/mL), streptomycin (100 μg/mL), and 10% fetal bovine serum (FBS) in an atmosphere of 95% air and 5% CO_2_ at 37 °C. When indicated, 4-PBA (3 mM) or 3-MA (5 mM) was added 1 h before the SH treatment.

### 4.3. Cell Viability Measurement

The CCK-8 assays were applied to detect cell viability. The cells were seeded in 96-well plates and treated with the indicated treatments for the indicated time points. At the end of the experiments, CCK-8 were added to each well following the kit assay protocol.

### 4.4. Cell Cycle Analysis

U87 and SF767 cells were incubated with different concentrations of SH for 24 h. Cells were harvested, washed three times with phosphate-buffered saline (PBS), and then fixed with ice-cold 70% (*v*/*v*) ethanol for 16 h at 4 °C. Prior to the analysis, cells were washed with PBS, suspended in 1 mL of cold PI solution (50 μg/mL PI and 100 μg/mL RNase A) and incubated for 20 min in the dark at 37 °C. Samples were examined using a FACScan flow cytometer (Becton Dickinson, Franklin Lakes, NJ, USA).

### 4.5. In Vitro Migration and Invasion Assays

A scratch wound healing assay was performed to investigate the migration of human glioblastoma cells, as previously described [70]. After a scratch was made with a 10 μL pipette tip, the wounded U87 or SF767 monolayers were washed twice with PBS to remove non-adherent cells and further treated with the indicated agents for 24 h. Wound healing was quantified and photographed. Moreover, the migratory capacity of human glioblastoma cells was assessed using Transwell migration assays, as described previously [70]. Cell suspensions (1 × 10^5^ cells) containing the indicated agents were seeded into the upper chambers with serum-free DMEM-H. DMEM-H containing 10% FBS were added to the lower chambers. After 24 h, the cells that had migrated to the lower surface of the filter were fixed with 4% paraformaldehyde, stained with 0.5% crystal violet, and then photographed. Additionally, invasion assays were conducted using Transwell inserts coated with matrigel, as described previously [70]. Cell suspensions (1 × 10^5^ cells) containing the indicated agents were seeded into the upper chambers with serum-free DMEM-H. DMEM-H containing 10% FBS were added to the lower chambers. After 24 h, the cells that had invaded were fixed with 4% paraformaldehyde for 20 min at room temperature, stained with 0.5% crystal violet for 10 min at room temperature, and then photographed. The mean values of six fields from *n* = 3 experiments were used to calculate the average number of cells.

### 4.6. Quantitative Real-Time PCR

Human glioblastoma U87 and SF767 cells were incubated with or without SH (0.125 and 0.25 mM) for 24 h, and their total RNAs were extracted from cells using the TRIzol reagent (Life Technologies, Grand Island, NY, USA). Reverse transcription was performed using a ReverTra Ace qPCR RT kit (Toyobo, Osaka, Japan). The sequences of specific primers for each gene are [71]: *MMP-9*, 5′-ATGGAGCTGGAATTGGATGC-3′ (forward), 5′-CTAGCCTAGATATCTGTCCT-3′ (reverse); *MMP-2*, 5′-TACACCTATACCAAGAACTTCCG-3′ (forward), 5′-TGTCCGCCAGATGAACCG-3′ (reverse); and *GAPDH*, 5′-TCACTGCCACCCAGAAGA-3′ (forward), 5′-TACCAGGAAATGAGCTTGAC-3′ (reverse). Real-time PCR was carried out with a QuantiTect SYBR Green RT-PCK Kit (QIAGEN, Hilden, Germany) using an ABI Prism 7000 Sequence Detector (Applied Biosystems, Darmstadt, Germany). Relative levels of the *MMP-2/-9* mRNAs were normalized to the *GAPDH* mRNA. The experiment was performed three times.

### 4.7. Co-IP Analysis

Cell lysates were pre-cleared by an incubation with Protein A + G Agarose and IgG (Beyotime, Nantong, China) at 4 °C for 2 h on a rotating platform. Centrifuged supernatants were incubated with the anti-IκB-α primary antibody or IgG at 4 °C overnight and further mixed with Protein A + G Agarose at 4 °C for 3 h. Protein A + G Agarose beads obtained by centrifugation were washed with Cell Lysis Buffer (Beyotime, Nantong, China) and re-suspended in 2 × sodium dodecyl sulfate (SDS) loading buffer (Beyotime, Nantong, China). Finally, Western blots were performed.

### 4.8. Confocal Microscopy

Immunofluorescence staining. After an incubation with SH (0.25 mM) for 24 h, U87 and SF767 cells were fixed with 4% paraformaldehyde, permeabilized in 0.5% Triton X-100 and blocked with 5% bovine serum albumin (BSA). After blocking, cells were stained with an anti-NFκB p65 antibody at 4 °C for 16 h. After three washes with PBS, cells were stained by FITC-conjugated anti-mouse IgG antibody for 1 h at room temperature and then stained with DAPI for 10 min at room temperature. Images were captured using a laser scanning confocal microscope (Leica, Heidelberg, Germany).

MDC was used as a specific autophagolysosome marker to observe the autophagy process. When the cells reached 75% confluence, they were incubated with the indicated treatments for 24 h. After two washes with PBS, AVs of the cells were stained with 50 μM MDC for 15 min at 37 °C in the dark. At the end of the incubation, cells were washed with PBS three times and photographed with a laser scanning confocal microscope.

### 4.9. Plasmids and siRNA Transfection

For NFκB p65 (human) overexpression, the gene (NM_021975.3) was cloned into the pcDNA3.1 (+) expression vector (GenePharma, Shanghai, China). pcDNA3.1 (+), an empty vector, was applied as a control. Cells were transfected with 2 μg/mL plasmid according to the manufacturer’s protocols. Medium supplemented with Lipofectamine™ 2000 was replaced with DMEM-H containing 10% FBS after 5 h. The CHOP (human) and ATG5 (human) siRNAs, as well as a control siRNA, were purchased from Santa Cruz Biotechnology. Cells were transfected with 150 nM siRNA, and the medium supplemented with Lipofectamine™ 2000 was replaced with fresh cell culture medium after 5 h. Subsequently, cells were incubated with the indicated treatments and subjected to further experiments.

### 4.10. Measurement of Intracellular Ca^2+^ Concentrations

[Ca^2+^]i was measured as described previously [72]. Briefly, U87 and SF767 cells were seeded on 24-well plates at a density of 5 × 10^4^ cells/mL. At the end of the treatment, cells were obtained and incubated with culture medium supplemented with 5 μmol/L Fura-2 AM (Beyotime, Nantong, China) at 37 °C for 45 min. Subsequently, cells were washed and resuspended with cold PBS supplemented with 0.2% BSA. Cells were incubated at 37 °C for another 5 min immediately before detection. [Ca^2+^]i was monitored using a fluorescence spectrophotometer (SpectraFluor, Tecan, Sunrise, Austria) at alternating excitation wavelengths ranging between 340 and 380 nm with an emission wavelength of 510 nm.

### 4.11. ELISA

TNF-α and IL-1β levels were investigated using a Quantikine ELISA Kit (R&D Systems, Shanghai, China), according to the manufacturer’s manual.

### 4.12. Western Blot

Cells were lysed on ice in Cell Lysis Buffer (Beyotime, Nantong, China) containing a complete protease inhibitor mixture (Roche Applied Science, Indianapolis, IN, USA) and phosphatase inhibitor (Beyotime, Nantong, China). Protein concentrations were measured with a BCA Kit (Beyotime, Nantong, China). Approximately ten to seventy micrograms of protein were separated by 6% to 15% SDS-polyacrylamide gel electrophoresis (PAGE), transferred, blocked, and blotted with primary antibodies, followed by an incubation with secondary antibodies. Afterwards, membranes were washed with Tris-buffered saline-Tween (TBST) three times, and the bands were monitored using chemiluminescence.

### 4.13. Immunohistochemistry

Briefly, tumor tissues embedded in paraffin were obtained from our previous experiments [26]. Samples were sectioned, stained with hematoxylin (Sinopharm Chemical Reagent Co., Ltd., Beijing, China) and antibodies against PCNA, p21, MMP-2, MMP-9, NFκB p65, CHOP, LC3B, vimentin, Snail or Slug. The primary antibodies were applied at 1:200 for PCNA, 1:200 for p21, 1:400 for MMP-2, 1:400 for MMP-9, 1:1000 for NFκB p65, 1:200 for CHOP, 1:400 for LC3B, 1:200 for vimentin, 1:200 for Snail and 1:200 for Slug. Finally, sections were mounted with DPX mounting media (Sigma-Aldrich), and images were obtained using a microscope (Leica, Wetzlar, Germany).

### 4.14. Statistical Analysis

The statistical analysis between two groups was performed using unpaired Student’s *t*-tests, and differences between various groups were analyzed using ANOVA and the least significant difference (LSD) *post hoc* test. Data were presented as means ± SEM. *p* < 0.05 was considered to be statistically significant.

## Abbreviations

SH	Sinomenine hydrochloride
LC3B	Microtubule-associated protein light chain 3B
3-MA	3-methyladenine
MDC	Monodansylcadaverine
ATG5	Autophagy-related 5
siRNA	Small interfering RNA
MMP	Matrix metalloproteinase
ER	Endoplasmic reticulum
EMT	Epithelial-mesenchymal transition
CHOP	C/EBP homologous protein
4-PBA	4-phenylbutyric acid
ECM	Extracellular matrix
UPR	Unfolded protein response
TNF-α	Tumor necrosis factor-α
IL-1β	Interleukin-1β
PERK	Eukaryotic translation initiation factor 2α kinase 3
GRP78	ER intraluminal 78 kDa glucose-regulated protein
eIF2α	Eukaryotic translation initiation factor 2α
AVs	Autophagic vacuoles
